# RXLR effector gene *Avr3a* from *Phytophthora sojae* is recognized by *Rps8* in soybean

**DOI:** 10.1111/mpp.13190

**Published:** 2022-02-12

**Authors:** Geneviève Arsenault‐Labrecque, Parthasarathy Santhanam, Yanick Asselin, Benjamin Cinget, Amandine Lebreton, Caroline Labbé, François Belzile, Mark Gijzen, Richard R. Bélanger

**Affiliations:** ^1^ Department of Phytology Université Laval Québec Québec Canada; ^2^ Agriculture and Agri‐Food Canada London Ontario Canada

**Keywords:** *Avr* gene, CRISPR/Cas9, effectors, oomycete, Phytophthora root rot, plant–pathogen interaction *Rps* gene

## Abstract

The use of resistance genes in elite soybean cultivars is one of the most widely used methods to manage *Phytophthora sojae*. This method relies on effector‐triggered immunity, where a Resistant to *P. sojae* (*Rps*) gene product from the plant recognizes a specific effector from the pathogen, encoded by an avirulence (*Avr*) gene. Many *Avr* genes from *P*. *sojae* have been identified in the last decade, allowing a better exploitation of this type of resistance. The objective of the present study was to identify the *Avr* gene triggering immunity derived from the soybean resistance gene *Rps8*. The analysis of a segregating F_2_ progeny coupled with a genotyping‐by‐sequencing approach led to the identification of a putative *Avr8* locus. The investigation of this locus using whole‐genome sequencing data from 31 isolates of *P*. *sojae* identified *Avr3a* as the likely candidate for *Avr8*. Long‐read sequencing also revealed that *P*. *sojae* isolates can carry up to five copies of the *Avr3a* gene, compared to the four previously reported. Haplotype and transcriptional analyses showed that amino acid changes and absence of *Avr3a* transcripts from *P. sojae* isolates caused changes in virulence towards *Rps8*. Functional analyses using CRISPR/Cas9 knockout and constitutive expression demonstrated that *Rps8* interacted with *Avr3a*. We also showed that a specific allele of *Avr3a* is recognized by *Rps3a* but not *Rps8*. While *Rps3a* and *Rps8* have been previously described as closely linked, this is the first report of a clear distinction hitherto undefined between these two resistance genes.

## Graphical abstract

The RXLR effector coded by a specific allele of the avirulence gene *Avr3a* from *Phytophthora sojae* (causing Phytophthora root rot) triggers immunity derived from the soybean resistance gene *Rps8*.

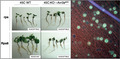

## 1 INTRODUCTION

The oomycete *Phytophthora sojae* is a soilborne pathogen causing Phytophthora root and stem rot (PRR), one of the major threats to the ever‐expanding soybean crop. In poorly drained fields with a history of the disease, yield losses can reach 100% when susceptible cultivars are planted (Dorrance, 2018). Over the last two decades, the sum of economic losses attributed to PRR in the United States has been higher than $5000 per hectare, making it among the most important diseases affecting soybean in the country (Bandara et al., 2020). Worldwide, the disease is causing annual yield damages of up to $2 billion (Tyler, 2007). To manage the pathogen, seed treatments, improved drainage, and host resistance are being used.

Host resistance has long been recognized as a very effective way to manage the disease as numerous sources of quantitative and qualitative host resistance have been identified (Dorrance, 2018). Quantitative disease resistance, also called partial resistance, is conferred by different quantitative trait loci (de Ronne et al., 2020). This type of resistance is known to be particularly effective when used in combination with qualitative resistance, named race‐specific resistance, or vertical resistance, which is conditioned by single dominant genes called Resistant to *P*. *sojae* (*Rps*) (Dorrance et al., 2003). This type of resistance, giving an immune type of response, is the most widely used as it can confer complete resistance against PRR. Controlled by a single gene, it is also easier to select phenotypes during breeding and facilitates their introgression into elite cultivars. Soybean is indeed a rich source of race‐specific resistance as nearly 30 *Rps* genes have been identified and mapped to nine chromosomes since the 1950s (Zhong et al., 2020). From this catalogue of genes, six have been successfully introgressed into commercial soybean varieties, namely *Rps1a*, *Rps1b*, *Rps1c*, *Rps1k*, *Rps3a*, and *Rps6* (Abeysekara et al., 2016; Sugimoto et al., 2012).

The resistance conferred by an *Rps* gene relies on the traditional gene‐for‐gene concept where a relationship exists between host resistance genes and pathogen virulence factors (Flor, 1971). Under this concept, *Rps* genes in the host encode or are predicted to encode specific nucleotide‐binding, leucine‐rich repeat immune receptors that can recognize specific effectors that typically contain N‐terminal RXLR (Arg‐any amino acid‐Leu‐Arg) and EER (Glu‐Glu‐Arg) motifs produced by avirulence (*Avr*) genes in the pathogen. The recognition of these avirulence factors will lead to effector‐triggered immunity in the host plant (Białas et al., 2018; Dodds & Rathjen, 2010). Conversely, the pathogen can avoid recognition conferred by *Rps* genes through various mutations, insertions, deletions, or altered expression of its corresponding *Avr* genes (Tyler & Gijzen, 2014).

This dynamic interplay between effectors and receptors has led to the evolution of more than 200 different pathotypes of *P*. *sojae*, as more *Rps* genes have been deployed commercially (Stewart et al., 2014). To better exploit the efficacy of each *Rps* gene in elite soybean cultivars, it is then crucial to have a good understanding of each corresponding *Avr* gene and its haplotypic diversity. To date, several *P*. *sojae Avr* genes have been identified, including *Avr1a*, *Avr1b*, *Avr1c*, *Avr1d*, *Avr1k*, *Avr3a/5*, *Avr3b*, *Avr3c*, and *Avr4/6* (Dong et al., 2009; Dong, Yin, et al., 2011; Dong, Yu, et al., 2011; Dou et al., 2010; Na et al., 2013, 2014; Qutob et al., 2009; Shan et al., 2004; Song et al., 2013). Recently, it was also found that for seven of these *Avr* genes recognized by the seven most common *Rps* genes, genomic signatures can be used as accurate predictors of phenotypes (Arsenault‐Labrecque et al., 2018). Subsequently, a molecular assay that reveals the avirulence allele of these seven *Avr* genes was developed in order to diagnose with precision the pathotypes of *P*. *sojae* isolates (Dussault‐Benoit et al., 2020).

While these recent developments will enable growers to better fight PRR, the constant adaptation of *P*. *sojae* makes the average durability of *Rps* genes within commercial soybean varieties to be approximately 8–20 years (Grau et al., 2004; Schmitthenner, 1985). This leads to the constant need of deploying new *Rps* genes and thereby subsequent identification of associated *Avr* genes from *P*. *sojae*. One of these resistance genes is *Rps8*, which maps on chromosome 13, close to the *Rps3* locus (Burnham et al., 2003; Dorrance et al., 2016; Gordon et al., 2006; Sandhu et al., 2005). As a matter of fact, *Rps3a* and *Rps8* have yet to be dissociated clearly (Gunadi, 2012) while other reports indicate that *Rps5* could also be linked to *Rps3a* (Dong et al., 2011). *Rps8* is of particular interest as it showed potential for management of *P*. *sojae,* notably in the United States and Brazil (Costamilan et al., 2012; Dorrance et al., 2016). These two countries account for more than 70% of global soybean production and both struggle with PRR problems.

The objective of this study was to identify *Avr8*, presumably an RXLR effector, from *P*. *sojae* leading to an immune response from the plants bearing *Rps8*. We first identified the *Avr8* locus via genetic mapping of this locus within a segregating F_2_ population of *P*. *sojae* isolates. These were tested for virulence in a hydroponic assay and genotyped via a genotyping‐by‐sequencing (GBS) approach. By taking advantage of exhaustive whole‐genome sequencing (WGS) data for a collection of *P*. *sojae* from a previous study (Arsenault‐Labrecque et al., 2018), it was possible to identify a single gene at the *Avr8* locus, namely *Avr3a*, that interacts with *Rps8*. The functional interaction of the product of the *Avr3a* gene with *Rps8* was first confirmed via genome editing with CRISPR/Cas9 and showed a clear compatibility between plants carrying *Rps8* and isolates lacking the *Avr3a* gene. In parallel, constitutive expression experiments validated the interaction between *Rps8* and *Avr3a* as its expression triggered an incompatible interaction with plants carrying *Rps8*. Nanopore long‐read sequencing also highlighted the presence of a fifth copy of *Avr3a* from isolates carrying the *Avr3a* ^45C^ allele, which is the one recognized by *Rps8*.

## 2 RESULTS

### 2.1 Localization of the *Avr8* region

**FIGURE 1:**
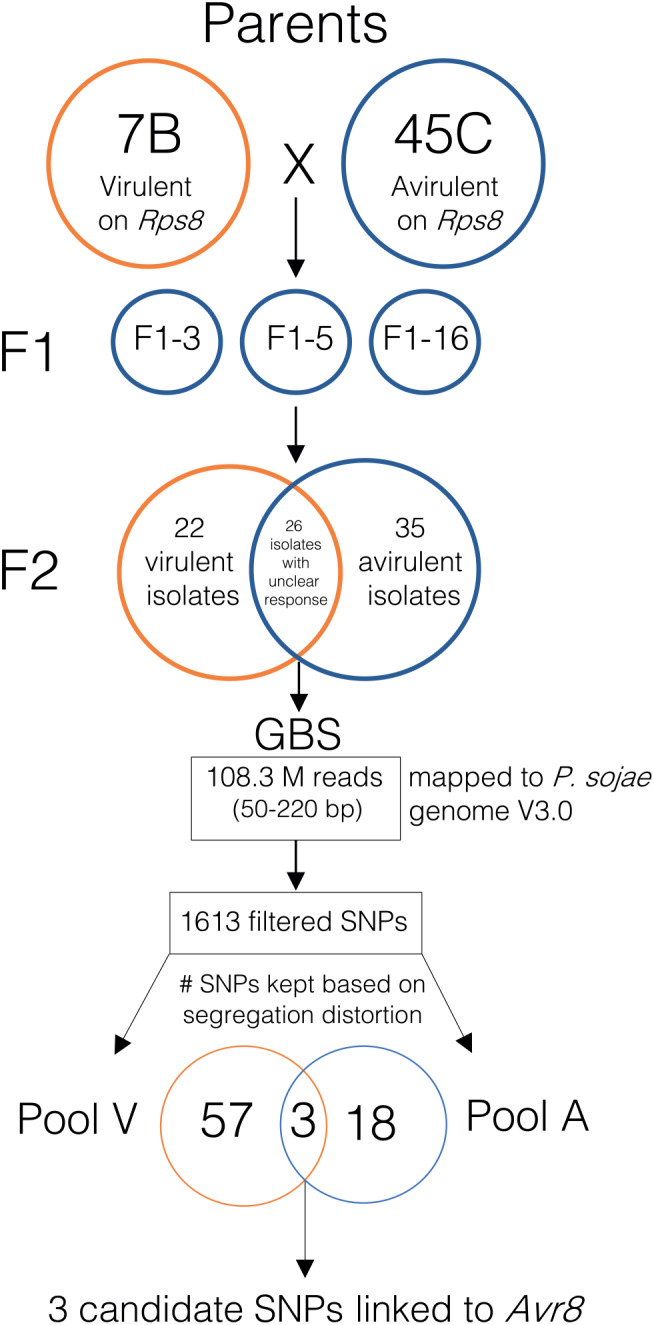
Genetic mapping of the *Avr8* locus in *Phytophthora sojae* in a segregating F_2_ population. Single‐nucleotide polymorphisms (SNPs) showing a marked allelic contrast between virulent and avirulent isolate were deemed to mark the *Avr8* locus

From the crosses of *P*. *sojae* isolates 45C (avirulent on *Rps8*) and 7B (virulent on *Rps8*), three F_1_ hybrids were obtained, and the phenotypic assay revealed that they were all avirulent on *Rps8* (Figure 1). Self‐fertilization of each of these F_1_ hybrids led to a total of 83 F_2_ progenies. Based on the hydroponic phenotyping assay, 35 F_2_ progenies were determined to be avirulent on *Rps8* while 22 were virulent. The remaining 26 F_2_ isolates showed intermediate responses towards *Rps8* and/or the susceptible control plants.

The 83 F_2_ progenies were genotyped using a GBS approach. A total of 110.8 million reads (50–220 bp in length) for an average of 1.34 million reads per isolate were obtained, and 97.7% of these reads were successfully mapped to the *P. sojae* reference genome (v. 3.0). After retaining positions with a coverage depth equal to or higher than 10, these reads covered approximately 2.5% of the whole genome and 29,053 single‐nucleotide polymorphisms (SNPs) were successfully called from these data (with minor allele frequency >0.008). Using SnpEff, it was found that 35% of those SNPs were in coding regions and 51% in the immediate vicinity of a coding region (1000 bp upstream or downstream of a coding region), with the remaining 14% located in intergenic regions. Among SNPs present in the coding regions, 63% induced nonsynonymous mutations. After filtering and imputation of missing data, a set of 1613 SNPs was retained (Figure 1).

**FIGURE 2:**
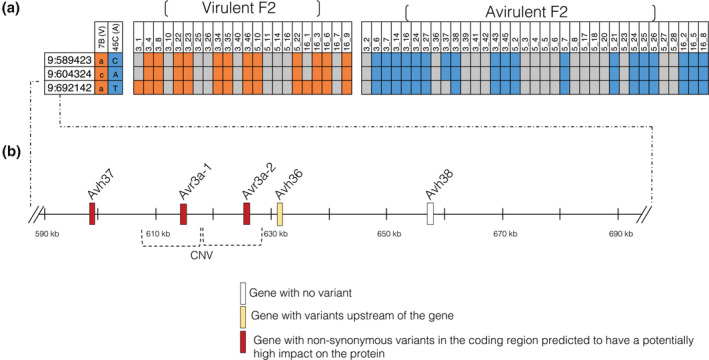
*Avr8* locus of *Phytophthora sojae* defined in this study. (a) Genotypes of F_2_ isolates for single‐nucleotide polymorphisms (SNPs) linked to *Avr8*. Orange and blue boxes represent homozygous genotypes and grey boxes represent heterozygous genotypes. (b) Overview of the potential impact of variants inherited from the virulent parent 7B on genes encoding RXLR effectors located in the *Avr8* region

Two pools of individuals were created based on their phenotypic reaction on *Rps8*: a virulent (V) pool and an avirulent (A) pool. For each SNP, the frequency of each allele was estimated in the entire F_2_ population, as well as in pools V and A. For each SNP in each pool, χ^2^ tests were performed to assess segregation distortion from the entire F_2_ population. When a SNP locus presented a significant segregation bias in both pools and the genotype of the favoured allele matched the parent with the corresponding phenotype, it was considered as potentially linked to *Avr8*. In pool V, a total of 60 SNPs inherited from the virulent parent presented a segregation bias while 21 SNPs in pool A, inherited from the avirulent parent, had a significant segregation distortion. From these selected SNPs, three consecutive SNPs were present in both pools and exhibited *p* values and genotype distributions that met all expected criteria for an avirulence locus. The allele frequencies of the candidate alleles for each SNP in each pool are presented in Table 1 and the genotype of each F_2_ isolate for each of these variants is shown in Figure 2a.

**TABLE 1:** Candidate single‐nucleotide polymorphisms (SNPs) linked to *Avr8* in *Phytophthora sojae*

SNP	Position	Allele frequency pool V^a^	Allele frequency pool A
1	PHYSOscaffold_9:589,423	0.76^b^	0.89
2	PHYSOscaffold_9:604,324	0.76	0.89
3	PHYSOscaffold_9:691,142	0.81	0.87

^a^ V: 7B (virulent parent); A: 45C (avirulent parent).

^b^ Frequency of the favoured allele at the SNP loci showing the greatest degree of segregation distortion (χ^2^ test, *p* < 0.05 in both pools).

The region circumscribed by the candidate SNPs is a gene‐rich region with a total of 34 genes, from which five have been previously characterized as either *Avr* genes (*Avr3a‐1* and *Avr3a‐2*) or effectors based on the presence of RXLR motifs (*Avh37*, *Avh36*, and *Avh38*).

### 2.2 RXLR effector candidates for *Avr8*

From the catalogue of variants called using WGS data for 31 isolates of *P*. *sojae*, including parents 7B and 45C, variants located in the *Avr8* locus and for which the virulent parental isolate 7B carried the alternative allele were extracted. A total of 252 SNPs and indels smaller than 50 bp, two deletions of more than 50 bp, and one copy number variation were found in this region (Table S1).

Using SnpEff, it was revealed that 75% of these variants were nonsynonymous and could potentially affect 28 out of 34 genes (of which four are predicted to encode RXLR effectors as presented in Figure 2b) by being located within the gene coding region or within 1000 bp upstream or downstream of these genes. Of these nonsynonymous variants, 60 were located directly in the coding regions of 15 genes, including the predicted RXLR effector gene *Avh37* (four variants) and two copies of the *Avr3a* gene (*Avr3a‐1* and *Avr3a‐2*; 16 variants). Five variants out of these 60 were also predicted to have a potentially high impact on the proteins coded by five different genes, including *Avh37* (frameshift insertion) and *Avr3a‐1* and *Avr3a‐2* (loss of stop codon for both genes). Furthermore, the copy number variation region detected in the parents included both copies of *Avr3a‐1* and *Avr3a‐2* represented in the reference genome of *P*. *sojae*, which are two exact copies of the same gene. The two deletions of 276 bp detected in the virulent parent were also located 1.1 kb upstream of *Avr3a‐1* and *Avr3a‐2*. A summary of the impact of the variants on RXLR effector genes at the *Avr8* locus is presented in Figure 2b and variants associated with genes encoding RXLR effectors present at the *Avr8* locus are shown in Table 2. These results led us to consider *Avh37* and *Avr3a* as the most promising candidates for *Avr8*.

**TABLE 2:** Summary of the variants affecting gene translation and encoding predicted RXLR effectors in the *Avr8* locus of *Phytophthora sojae*

Predicted RXLR effector	Position on PHYSOscaffold_9	Polymorphisms from virulent parent 7B
*Avh37*	598,793–598,248	7 variants upstream of the gene including 2 insertions of 6 and 25 bp 2 missense mutations 1 in‐frame insertion 1 frameshift insertion
*Avr3a*	615,105–615,440 (*Avr3a‐1*)	9 variants upstream of the gene including 1 deletion of 276 bp and 3 insertions of 10, 11, and 26 bp 14 missense variants 1 in‐frame insertion Loss of stop codon Copy number variation
	625,907–626,242 (*Avr3a‐2*)	
*Avh36*	631,330–631,530	2 variants upstream of the gene
*Avh38*	657,499–657,924	No variant

**FIGURE 3:**
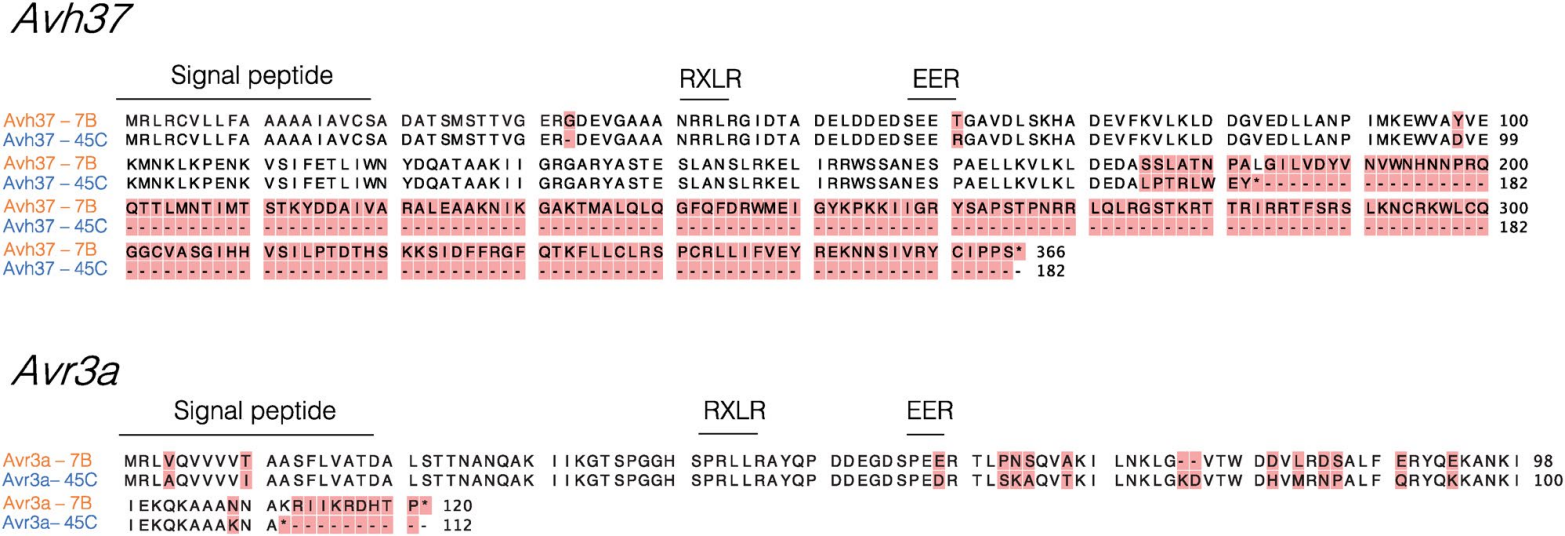
Predicted amino acid sequences of *Phytophthora sojae Avr8* candidate genes, based on alleles from parent 7B (virulent on *Rps8*) and 45C (avirulent on *Rps8*). Signal peptides and RXLR and EER motifs are shown, and polymorphic residues among the two different alleles are highlighted with a red background for *Avh37* and *Avr3a* genes

Based on the polymorphisms presented in Table 2, predicted amino acid sequences of the different alleles of the two candidate genes *Avh37* and *Avr3a* are shown in Figure 3. For both genes, amino acid changes were found in the N‐terminal portion, as well as in the C‐terminal portion. In the N‐terminal portion, amino acid changes altered the EER motif of the virulent alleles for both genes. Two amino acid changes were also located in the signal peptide for *Avr3a*. The frameshift insertion in the C‐terminal portion of *Avh37^7^* ^B^ led to two proteins of different length as the stop codon from the 45C allele was lost. For *Avr3a*, the stop codon present in the 45C allele was also lost due to a SNP.

### 2.3 Transcript analysis of candidate genes

Expression levels of candidate genes *Avh37* and *Avr3a* in five different isolates of *P. sojae* were compared to determine whether transcript abundance could be associated with virulence phenotypes; results are shown in Table 3 (original image from gel electrophoresis is presented in Figure S1). For *Avh37*, the transcript was present in every isolate studied regardless of the phenotype on *Rps8* or the allele of the gene carried by these isolates. For *Avr3a*, transcriptional analysis showed that expression of the gene occurred in the three isolates avirulent on *Rps8* and that possessed the *Avr3a* allele from the avirulent parent 45C. On the other hand, *Avr3a* mRNA was not detected in the two virulent isolates tested and carrying the allele of isolate 7B, which is virulent on *Rps8*.

**TABLE 3:** Reverse transcription PCR analysis of gene expression of candidate genes *Avh37* and *Avr3a* in *Phytophthora sojae* isolates showing contrasting phenotypes on soybean plants carrying *Rps8*

Isolate	Virulence *Rps8* ^a^	*Avh37*		*Avr3a*	
		Sequence^b^	mRNA^c^	Sequence	mRNA
F2‐3‐7	A	45C	+	45C	+
45C	A	45C	+	45C	+
8‐5‐7	A	45C	±	45C	+
Race 7	V	7B	+	7B	−
2012_70	V	7B	+	7B	−

^a^ A: avirulent; V: virulent.

^b^ Two different sequences, according to the sequence from the representative strains 7B (virulent parent) and 45C (avirulent parent).

^c^ (+), mRNA detected; (−), mRNA not detected. Transcriptional analysis was performed by reverse transcription PCR on mRNA isolated from infected root tissues 5 dpi.

### 2.4 Interaction of effectors encoded by candidate genes with *Rps8*

To test the interaction of Avr proteins encoded by candidate genes *Avh37* ^45C^ and *Avr3a* ^45C^ with the soybean *Rps8* gene product, two different *P*. *sojae* transformations were performed. To see if the absence of transcript from each candidate gene caused gain of virulence in the presence of *Rps8*, CRISPR/Cas‐9‐mediated genome editing was used to knock out the two candidate genes independently in the *P*. *sojae* isolates showing avirulence on *Rps8*. Constitutive expression of the candidate genes in a *P*. *sojae* isolate virulent on *Rps8* was also performed to determine if its expression would trigger a defence response from plants carrying *Rps8*. Regarding the CRISPR/Cas9 transformation, 36 transformants were screened and sequenced and one stable transformant was obtained for *Avr3a*, while 48 transformants were screened and sequenced but no stable transformants were generated for *Avh37*. For the constitutive expression, two stable transformants were obtained for *Avr3a* and three for *Avh37*. Phenotyping assays were conducted for each of these transformants.

**FIGURE 4:**
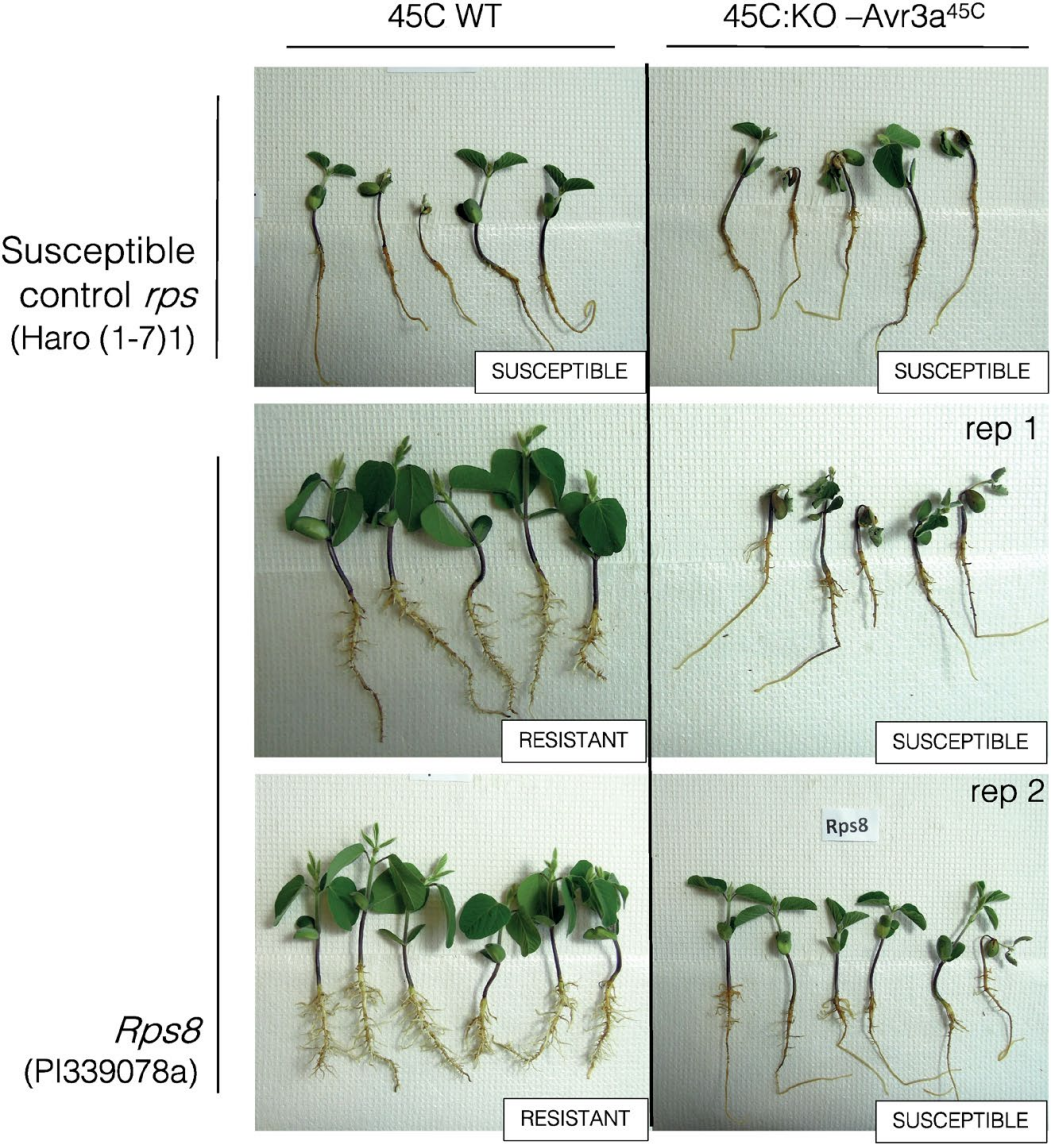
Virulence phenotypes of *Phytophthora sojae* wild‐type (45C) and *Avr3a* knockout (KO) strains

The stable transformant obtained from CRISPR/Cas9 transformation targeting the *Avr3a* ^45C^ gene presented a single‐nucleotide deletion causing a mutation in the dEER (Asp‐Glu‐Glu‐Arg) motif of the protein and leading to a truncated protein before the predicted effector domain containing the W‐like motif near the C‐terminal end (see Figure S2). Phenotyping results of the wild‐type isolate 45C (45C WT) and the *Avr3a* ^45C^ knockout isolate (45C:KO‐*Avr3a* ^45C^) are shown in Figure 4 and susceptibility scores can be found in Figure S3. As expected, the WT isolate 45C was avirulent in the presence of *Rps8*. By contrast, inoculation with the *Avr3a* ^45C^ knockout isolate led to a phenotype of virulence on plants carrying *Rps8*. As shown in Figure 4, plants containing *Rps8* showed resistance in both the aerial and root parts when inoculated with the WT isolate 45C, compared to the susceptible control plants Haro (1‐7)1. For their part, plants containing *Rps8* showed a compatible reaction when inoculated with the *Avr3a* ^45C^ knockout isolate, thus indicating that the lack of *Avr3a* ^45C^ gene resulted in a gain of virulence in the presence of *Rps8*.

**FIGURE 5:**
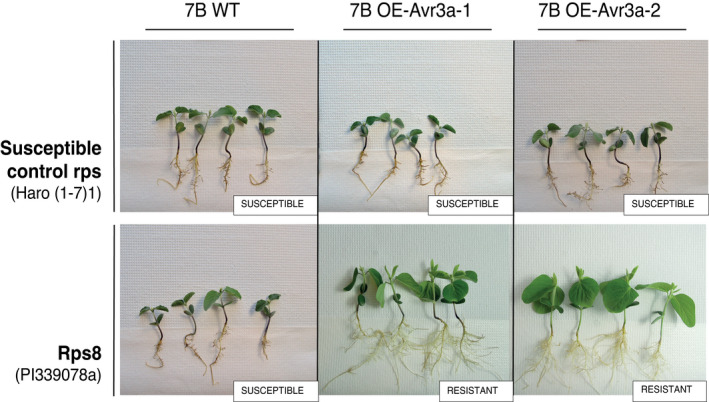
Virulence phenotypes of *Phytophthora sojae* wild‐type (7B WT) and *Avr3a* ^45C^ overexpression (OE) strains

With respect to the constitutive expression of *Avr3a* ^45C^, transcript analysis confirmed its expression in transformed isolates 7C OE‐*Avr3a*‐1, ‐2, and ‐3, while it went undetected in WT isolate 7B (Figure S4). Phenotyping results of the WT isolate 7B and the two transformants expressing *Avr3a* ^45C^ are shown in Figure 5 (susceptibility scores are found in Figure S3). The WT isolate 7B was virulent in the presence of *Rps8* on control plants Haro (1‐7)1, as anticipated. In contrast, transformed isolates expressing *Avr3a* ^45C^ were avirulent on plants containing *Rps8*, demonstrating that constitutive expression of *Avr3a* ^45C^ in *P*. *sojae* isolate 7B causes gain of avirulence in the presence of *Rps8*.

**FIGURE 6:**
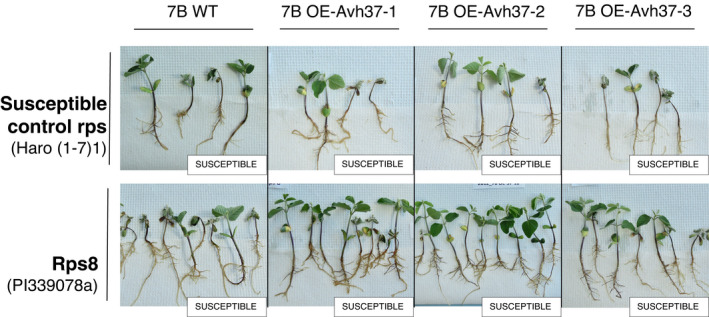
Virulence phenotypes of *Phytophthora sojae* wild‐type (7B WT) and *Avh37* ^45C^ overexpression (OE) strains

For candidate gene *Avh37*, transcript analysis was performed on WT isolate 7B (virulent in the presence of *Rps8*) and the three stable transformants obtained (7B OE‐*Avh37*‐1, ‐2, and ‐3) from constitutive expression of *Avh37* ^45C^. This analysis showed expression of the gene in transformed isolates and none in the WT isolate 7B (Figure S4). Phenotyping results of the WT isolate 7B and the three transformants expressing *Avh37* are shown in Figure 6 (susceptibility scores are found in Figure S3). As expected, the WT isolate 7B was virulent in the presence of *Rps8* and on susceptible control plants Haro(1‐7)1. Similar virulent responses were observed on plants inoculated with the transformants, showing that overexpression of *Avh37* did not cause an incompatible interaction with plants containing *Rps8*.

### 2.5 New definition of the *Avr3a* locus

To obtain a better definition of the *Avr3a* locus, Nanopore long‐read sequencing was performed for parental isolates 45C and 7B. To observe the inheritance pattern of the *Avr3a* region in the progeny, two F_2_ isolates (8‐3‐6, avirulent on *Rps8*, and 8‐3‐34, virulent on *Rps8*) were also sequenced. From the parental isolate 45C, a read was obtained with a length of 79,521 kb that carried five identical copies of the *Avr3a* gene. Each copy was embedded in a segment of 10.8 kb with four other genes, and this segment was repeated five times, as a result of tandem duplication (Figure 6). Reads from Illumina and Nanopore sequencing of isolate 45C were used to construct a new consensus sequence of the whole region (File S1). This sequence was used to align contigs obtained from de novo assembly of Nanopore reads of the F_2_ isolate 8‐3‐6 (avirulent on *Rps8*). This alignment showed that five identical copies of the *Avr3a* gene were also present in the avirulent F_2_ progeny. To define the *Avr3a* region in parental and F_2_ isolates virulent on *Rps8* (7B and 8‐3‐34), de novo assembly and alignment of contigs on the 45C new consensus sequences were performed, which demonstrated a clear deletion of four copies of the 10.8‐kb repetitive unit in both strains (Figure 6).

## 3 DISCUSSION

The use of *Rps* genes in elite soybean cultivars has proven to be an effective source of resistance against *P*. *sojae* in the last decades. However, the capacity of the pathogen to avoid recognition by *Rps* genes through various mutations of its corresponding *Avr* genes has led to the constant need of renewed sources of resistance. In turn, these require an in‐depth knowledge of the different mechanisms by which the pathogen succeeds in circumventing this resistance to maintain its virulence. The last two decades have been prolific in terms of the discovery of *P*. *sojae Avr* genes (Tyler & Gijzen, 2014) and the present study fits in this continuum with the identification of the *Avr3a* gene as encoding the effector interacting with *Rps8* from soybean. The presence of a fifth copy of this gene in a strain avirulent on *Rps8* also led to the definition of a new haplotype for this gene. This was made possible by using novel approaches such as an improved GBS technique (Sonah et al., 2013), a recently developed and accurate phenotypic assay (Lebreton et al., 2018), the CRISPR/Cas9 system for transformation of *P. sojae* (Fang et al., 2017), and the use of Nanopore long‐read sequencing.

The homothallic nature of *P*. *sojae* offers the advantage of facilitating the generation of F_2_ populations to follow the inheritance of virulence phenotypes. Many studies, including this one, have relied on this method for the identification of *P*. *sojae Avr* genes (Dong et al., 2009; Dong, Yin, et al., 2011; Dong, Yu, et al., 2011; Dou et al., 2010; Na et al., 2013, 2014; Qutob et al., 2009; Shan et al., 2004; Song et al., 2013). Downstream analysis of the F_2_ population requires a great set of high‐quality genotypic data to capture the precise genetic regions linked to the targeted phenotype. The existence of a repertoire of predicted and annotated RXLR effectors from *P*. *sojae* prompted the preselection of candidate effectors to accelerate the identification of some *Avr* genes (Dong et al., 2009; Dong, Yin, et al., 2011; Na et al., 2013). However, this strategy carries the risk of omitting potential effectors, as has been the case in initial attempts to identify *Avr1c* (Na et al., 2014). The advent of next‐generation sequencing (NGS) has given access to high‐throughput sequencing technologies that facilitate the search for *Avr* genes at the whole‐genome level. In our work, we further benefited from NGS technology by using an improved GBS method that yields high SNP coverage. This method uses restriction enzymes to reduce genome complexity and leads to extensive marker coverage across the entire genome (Elshire et al., 2011). The SNPs obtained from our sequencing data were indeed located in gene‐rich regions as 86% of the SNPs were found in coding regions or within 1000 bp of those regions. Previous studies in a variety of organisms simplified library preparation by pooling DNA samples based on phenotype prior to sequencing, in order to accelerate gene mapping (Abe et al., 2012; Austin et al., 2011; Na et al., 2014; Takagi et al., 2013). Given that the GBS approach enables a high level of multiplexing (Sonah et al., 2013), we were able to generate individual sequencing data for all 83 F_2_ isolates and pool genotype data afterwards. This allowed us to select candidate SNPs both on the basis of expected allelic frequencies in each pool and on the precise genotype expected from every F_2_ individual according to their phenotype. This brought a new level of precision to identify a locus of interest linked to the phenotype of avirulence on an *Rps* gene. This strategy including GBS sequencing and pooling of F_2_ isolates postsequencing led to the definition of the *Avr8* locus, which is a segment of 102.7 kb on PHYSOscaffold_9 where three RXLR effector genes are located among a total of 34 genes.

Once the locus for *Avr8* was defined, WGS data of 31 *P*. *sojae* isolates with a wide diversity of pathotypes allowed us to generate an exhaustive catalogue of SNPs and structural variations occurring in the region of interest. Because all *P*. *sojae Avr* genes identified to date are predicted to code for RXLR effector proteins, emphasis was placed on four genes encoding RXLR effectors found at the *Avr8* locus. Incidentally, for two of them, *Avr3a* and *Avh37*, many polymorphisms, including SNPs and structural variations, were present in the coding and promoter regions of the genes, making them prime candidates for *Avr8*.

Regarding the first candidate, *Avr3a*, the allele carried by the virulent parent, *Avr3a* ^7B^, possesses multiple amino acid changes that could impact the recognition of the protein by *Rps8*. Furthermore, no transcripts were detected from isolates carrying this allele. The multiple indels found in the promoter region of the gene, coupled with deletion of copies of the gene in virulent isolates carrying allele *Avr3a* ^7B^, could interfere with transcription, as has been hypothesized in previous work on *Avr3a* (Dong, Yu, et al., 2011; Shrestha et al., 2016). Therefore, the multiple polymorphisms associated with *Avr3a* and the differences in transcript abundance observed between isolates virulent and avirulent on *Rps8* prompted us to consider this gene as a good candidate for *Avr8*.

Regarding the second candidate, the allele associated with the virulent parent, *Avh37* ^7B^, contains an amino acid change that alters the dEER motif. This could possibly impact the translocation of the effector into the host cell, making it unrecognizable by *Rps8*. The frameshift insertion causing the loss of the stop codon leads to a sequence twice as long as the sequence of *Avh37* ^45C^, the allele present in the avirulent parent. Results from the transcript analysis showed that the gene was expressed in all isolates regardless of the phenotype on *Rps8*. For most of the *Avr* genes identified to date, a lower level of expression of the transcript or total loss of transcript are correlated with the phenotype of virulence. On the other hand, amino acid changes can also be sufficient to confer a gain of virulence (Dong et al., 2009; Dong, Yu, et al., 2011; Dou et al., 2010). For this reason and considering the possibility that the mutation in the dEER motif prevents recognition by *Rps8*, *Avh37* was further investigated to test its interaction with *Rps8*.

To determine the functionality of our candidate genes, the CRISPR/Cas9‐mediated genome editing method was first prioritized. Knockout of *Avr3a* in transformed isolate 45C:KO‐*Avr3a* ^45C^ demonstrated an evident gain of virulence in the presence of *Rps8*. Because the isolate 45C used for the knockout of *Avr3a* still expresses *Avh37* ^45C^, we concluded that *Rps8* plants interacted with only one gene product, *Avr3a*. Results of the overexpression of *Avr3a* ^45C^ in a *P*. *sojae* isolate virulent on *Rps8* and not expressing this gene also support the interaction between *Avr3a* and *Rps8* as the transformed isolates triggered resistance on plants carrying *Rps8*. Owing to our inability to obtain an *Avh3*7^45C^ knockout transformant via CRISPR/Cas9, we relied on overexpression of *Avh37* ^45C^ in a *P*. *sojae* isolate virulent on *Rps8* that does not normally express this gene to test the possible interaction with *Rps8*. The three transformed isolates expressing *Avh37* ^45C^ were as virulent on plants carrying *Rps8* as the WT isolate, supporting that *Avh37* does not induce resistance in plants carrying *Rps8*. These results confirmed that *Avr3a* alone yielded an incompatible interaction with *Rps8*.

**FIGURE 7:**
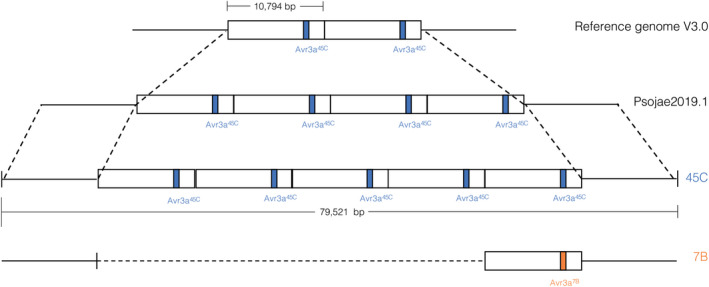
Representation of the copy number variation occurring in the *Avr3a* locus among *Phytophthora sojae* isolates. The red boxes represent the *Avr3a* gene. The *Avr3a* gene is embedded in a repetitive unit of 10,794 kb in every sequence. In the reference genome v. 3.0, based on Sanger sequencing of isolate P6497, two copies of the *Avr3a* ^45C^ are represented, while four copies are present in the assembly Psojae2019.1 based on the Nanopore sequencing of P6497. Nanopore sequencing of parental isolates revealed a fifth copy of *Avr3a* ^45C^ for isolate 45C (avirulent on *Rps8*) while isolate 7B (virulent on *Rps8*) carries only one copy of *Avr3a* ^7B^. The consensus sequence for 45C is based on a single read of 79,521 bp encompassing the whole region carrying the five copies of *Avr3a* and its flanking regions. Those flanking regions are identical among the different sequences represented here

The *Avr3a* gene encodes a well‐studied avirulence factor that has been extensively characterized in the past (Dong, Yu, et al., 2011; Qutob et al., 2009, 2013; Shrestha et al., 2016). In an attempt to get the highest resolution of the *Avr3a* region organization in our *P*. *sojae* population, we used Nanopore long‐read sequencing. The *Avr3a* region is indeed characterized by repetitive elements occurring in tandem arrays of clustered genes, making sequence assembly problematic, especially with short reads. Previous studies reported that the copy number of *Avr3a* varied from one to four, depending on the *P*. *sojae* isolate (Arsenault‐Labrecque et al., 2018; Qutob et al., 2009). Our long‐read sequencing data demonstrate that *P*. *sojae* can carry up to five copies of the *Avr3a* gene, as is the case in the parental isolate 45C Figure 7. Contigs obtained with reads of F_2_ isolates 8‐3‐6 (avirulent on *Rps8*) also support that these five copies are inherited by the F_2_ progeny. The fifth copy of the *Avr3a* gene defines a new haplotype for this *Avr* gene and emphasizes the role of this effector in pathogenicity, prior to its recognition by different resistance genes.

**FIGURE 8:**
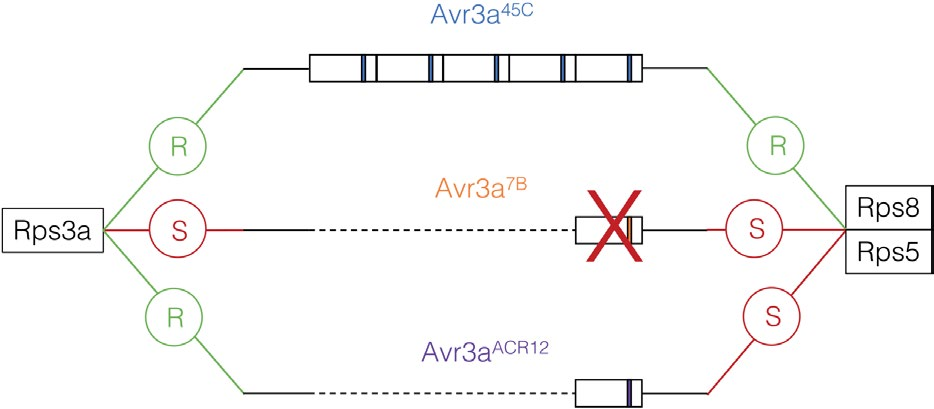
Schematic representation of the interaction of the different *Avr3a* alleles of *Phytophthora sojae* with soybean plants carrying *Rps3a* and *Rps8*. The letter in the circle represents the response from the plants carrying the corresponding *Rps* gene. R, resistant; S, susceptible. *P*. *sojae* isolates carrying the *Avr3a* ^45C^ allele possess multiple identical copies of the *Avr3a* gene while isolates carrying allele *Avr3a* ^7B^ or *Avr3a* ^ACR12^ carry only one copy. No transcripts of the *Avr3a* gene are expressed for isolates carrying *Avr3a* ^7B^

In our study, parental isolates used to identify *Avr8* were as virulent against *Rps8* as they were against *Rps3a*. Furthermore, 34 of the 35 isolates phenotyped in this study gave a similar response when tested against *Rps8* and *Rps3a* (see Table S2). Isolates with a phenotype of avirulence on *Rps3a*/*Rps8* possessed allele *Avr3a* ^45C^ while isolates virulent on *Rps3a* and *Rps8* possessed allele *Avr3a* ^7B^. From previous studies (Dong, Yu, et al., 2011; Qutob et al., 2009), these alleles correspond, respectively, to allele *Avr3a* ^P6497^, the allele recognized by *Rps3a* when expressed, and *Avr3a* ^P7064^, which is not expressed and associated to a phenotype of virulence on *Rps3a*. Only one isolate in our collection, ACR20, presented a differential reaction towards *Rps3a* and *Rps8*. Interestingly, that isolate carries a third allele of *Avr3a*, previously described as *Avr3a* ^ACR12^ (Table S2). This allele is expressed during infection but differs by two amino acids from *Avr3a* ^45C^ (K64P and A65S), a change that could possibly explain the differential responses of *Rps3a* and *Rps8* towards its gene product (Figure S4). It would indeed be plausible that *Rps3a* is able to recognize *Avr3a* ^ACR12^ while *Rps8* is not, as represented in Figure 8. The *Rps8* resistance gene was previously located on chromosome 13, as was *Rps3a*, and an allelism study suggested that *Rps8* and *Rps3a* are linked within this chromosome at ≥11.0 cM apart (Gordon et al., 2006; Gunadi, 2012). It has also been hypothesized that these two genes recognize the same *P*. *sojae* effectors or are closely related resistance genes because of the similar disease reaction pattern between *Rps3a* and *Rps8* from the 75 *P*. *sojae* isolates used in a previous study (Gunadi, 2012). The report in the present study of a third allele of *Avr3a* recognized differentially by *Rps3a* and *Rps8* supports that the two genes are clearly distinct and recognize different alleles of the same effector.

The fact that *Rps8* is able to recognize the product of the same *Avr* gene as *Rps3a* might explain in part the mitigated efficiency of *Rps8* towards *P*. *sojae* populations observed in the United States, even before its commercial deployment. In a survey from 2006, seven different pathotypes were observed among the *P*. *sojae* populations in Missouri and almost all isolates designated as races (pathotypes) 15, 17, 24, or 26. All four were highly virulent on soybeans carrying *Rps8* (Smith et al., 2006). Incidentally, these four isolates were distinct from the others by being also virulent on *Rps3a*. In a recent survey assessing the pathotype diversity of 873 *P*. *sojae* isolates from 11 states in the United States, *Rps8* presented higher efficiency as only 1.5% of all isolates were virulent on *Rps8* (Dorrance et al., 2016). Again, 85% of these isolates virulent on *Rps8* were also virulent on *Rps3a*. Interestingly, approximately 15% of the isolates from this study were virulent on *Rps3a* but not on *Rps8*, a phenotype that has not been encountered in our study. It would be interesting to retrieve those isolates that originate mostly from Illinois and to confirm their phenotypes and genotypes in order to investigate if an additional allele of *Avr3a* is recognized by *Rps8* but not *Rps3a*.

A previous study also demonstrated that *Avr3a* interacted with *Rps5* and that *Avr3a* and *Avr5* were allelic (Dong, Yu, et al., 2011). Surprisingly, when comparing the interaction of *Rps8* plants with the three different alleles of *Avr3a*, nothing could distinguish the response from *Rps8* and the responses from *Rps5* in this previous study (Table 4). The *Rps5* phenotype was first described in 1981 and has since been mapped on chromosome 18 and linked with *Rps4* and *Rps6* (Buzzell & Anderson, 1981; Sun et al., 2014). In another study that aimed to identify simple‐sequence repeat (SSR) markers linked to *Rps1* to *Rps6*, linkages were found for all but *Rps5*, due to a skewed genotypic segregation in the *Rps5*/*rps5* population (Demirbas et al., 2001). This complicates the understanding of the association among the *Rps3a*, *Rps5*, and *Rps8* genes. As opposed to *Rps3a*, *Rps5* does not seem to be genetically linked to *Rps8* despite its similar response to *Avr3a*. To distinguish the interaction of *Avr3a* with *Rps5* and *Rps8*, one would require *P*. *sojae* isolates that present distinctive phenotypes against each of the genes and investigate the genotypic differences between the isolates. That said, such distinct isolates are currently undefined to our knowledge.

**TABLE 4:** Virulence characteristics of *Phytophthora sojae* isolates on *Rps3a*, *Rps5*, and *Rps8* and haplotype analysis

Isolate	Virulence *Rps3a* ^a^	Virulence *Rps5* ^b^	Virulence *Rps8* ^a^	*Avr3a* allele	mRNA *Avr3a* c
P6497	A	A	A	45C/P6497	+
P7064	V	V	V	7B/P7064	−
P7074	V	V	V	7B/P7064	−
ACR20	A	V	V	ACR12	+

^a^ Virulence phenotypes observed in this study and in Dong, Yu, et al. (2011).

^b^ Virulence phenotypes obtained in Dong, Yu, et al. (2011).

^c^ Positive (+) or negative (−) for expression of *Avr3a* transcripts, as determined by reverse transcription PCR (Dong, Yu, et al., 2011).

The present study is the first to exploit the CRISPR/Cas9‐mediated genome editing method developed for *P*. *sojae* in order to knock out an *Avr* gene and confirm its interaction with an *Rps* gene (Fang et al., 2017). Previously, such knockouts were impossible to obtain with *P*. *sojae* because of the presence of nonhomologous end‐joining (Judelson, 1997; Tyler & Gijzen, 2014). Knockdown of *P*. *sojae* genes to identify avirulence factors has been mostly accomplished by gene silencing. However, this knockdown method leads to residual transcription and expression of the targeted gene that can leave intermediate responses from the plant (Fang & Tyler, 2016). By taking advantage of methods derived from NGS and CRISPR/Cas9‐mediated genome editing, this study identified *Avr3a* as the *Avr* gene recognized by *Rps8*, a resistance gene providing a new source of resistance for elite soybean cultivars (Abeysekara et al., 2016). In addition, overexpression of *Avr3a* in *P. sojae* transformants supported the fact that *Avr3a* led to an avirulent response when interacting with *Rps8*. Furthermore, long‐read sequencing brought a new insight for the *Avr3a* gene, with the discovery of a fifth copy of the gene in isolates avirulent on *Rps3a* and *Rps8*. A precise definition of the different haplotypes associated with the virulence of *P*. *sojae* towards *Rps8* will therefore make it possible to better manage the disease, especially in the context of the recent development of molecular tools (Dussault‐Benoit et al., 2020) that allow a tailored use of *Rps* genes based on a precise and rapid determination of pathotypes in soybean fields.

## 4 EXPERIMENTAL PROCEDURES

### 4.1 *P. sojae* isolates and plant materials

Detailed information about the 35 *P. sojae* isolates used in this study is listed in Table S2. Isolates were grown and maintained on V8 gellan gum medium at 28°C. For long‐term storage, pieces of mycelium grown on V8 agar for 2 weeks were transferred in 3 ml sterile deionized water in plastic sterile tubes. Tubes were kept at room temperature in the dark. Each isolate was characterized for the presence of *Avr* genes using a hydroponic assay in which zoospores are inoculated directly into the hydroponic nutrient solution (Lebreton et al., 2018). For each isolate, four plants from accessions Williams L83‐570 (*Rps3a*) and PI399073a (*Rps8*) and a susceptible control cultivar not carrying the appropriate *Rps* gene were tested. Phenotypic responses for resistance or susceptibility were recorded at 10 days postinoculation (dpi) and based on comparative responses of control resistant and susceptible cultivars.

### 4.2 Crossing of *P. sojae* isolates

To follow the inheritance of virulence towards *Rps8*, two *P*. *sojae* isolates from our collection, 45C (avirulent on *Rps8*) and 7B (virulent on *Rps8*), were crossed to obtain F_1_ and F_2_ progenies. Pieces of mycelium from long‐term storage were transferred on 20% V8 gellan gum (0.4%). The Petri dishes were placed in an incubator at 28°C. After 1 week of growth, mycelia from the two isolates were mixed together with a syringe and transferred on Petri plates containing crossing medium (2.5% clarified V8, 1.2% gellan gum supplemented with 10 μg/ml β‐sitosterol). The plates were left in the dark at room temperature. After 3 weeks, two plates were sliced using a sterile blade and blended together in a prechilled blender for 2 min with 50 ml of fridge‐cold sterile water. The mix was filtrated through four layers of sterile cheesecloth to remove agar and mycelium and the filtrate was transferred to a 50‐ml sterile conical tube. The mixture was kept on ice for 24 h to kill the residual mycelium. The filtrate was then centrifuged at 956 × *g*  for 3 min and the supernatant was removed. The pellet of oospores was washed three times with sterile water and centrifugation cycles. Oospores were transferred on water agar supplemented with penicillin at a concentration of 500 oospores per Petri dish and spread with a sterile loop. After three or four days, each germinating oospore was transferred individually using a 20‐gauge needle and grown on 20% V8 agar medium for subsequent DNA extraction.

### 4.3 F_1_ hybrid selection and F_2_ generation

DNA of each culture grown from pure isolated oospores was extracted using an adapted cetyltrimethylammonium bromide (CTAB) and chloroform/isoamyl alcohol extraction method followed by isopropanol precipitation. To determine F_1_ hybrid progeny from crosses of 45C × 7B, codominant DNA markers polymorphic between the parents were used to determine whether the individuals resulted from self‐fertilization or from outcrossing events between the parental isolates (F_1_). The polymorphism used to distinguish alleles from both parents was a deletion; isolate 7B has a deletion of 35 bp in the amplicon region while isolate 45C does not. Selected F_1_ hybrid isolates were then cultured individually on crossing medium in the dark at room temperature for self‐fertilization and generation of F_2_ individuals. Oospores produced from F_1_ individuals were isolated using the method described above and grown on 20% (vol/vol) V8 agar medium. All F_1_ and F_2_ individuals were phenotyped for their compatibility interaction with *Rps8* using the hydroponic assay developed by Lebreton et al. (2018) and cultivars PI399073a (*Rps8*) and susceptible control Haro (1‐7)1 (*rps*).

### 4.4 Segregant analysis of F_2_ populations

To find a region that co‐segregated with the phenotype of avirulence on *Rps8* and to identify potential candidate genes for *Avr8*, the F_2_ progenies were genotyped using a GBS approach. DNA from F_2_ individuals was extracted using the CTAB and chloroform/isoamyl alcohol method followed by isopropanol precipitation. DNA was quantified using a Qubit 4 fluorometer (Thermo Fisher Scientific) and concentrations were normalized to 10 ng/µl for library preparation. Sequencing libraries were prepared using the *Ape*K1 restriction enzyme following the protocol from Elshire et al. (2011), with minor modifications as described by Sonah et al. (2013). Single‐end sequencing of multiplex GBS libraries was performed on an Ion Proton sequencer (Thermo Fisher Scientific) at the Institut de Biologie Intégrative et des Systèmes (IBIS) of Université Laval, Quebec, Canada. Read processing, mapping to the *P*. *sojae* reference genome v. 3.0 (Tyler et al., 2006), variant calling, and genotyping were performed using the Fast‐GBS pipeline (Torkamaneh et al., 2017), using default parameters except for the depth of coverage (minimum of 10 supporting reads before a variant candidate was considered, instead of two). Variants were filtered using VCFtools (Danecek et al., 2011) with the following parameters: biallelic SNPs conserved only, maximum missing data (MaxMD) = 80%, and minimum minor allele frequency (MinMAF) = 0.2. Missing data were imputed using Beagle (Browning & Browning, 2016).

Polymorphisms detected were used to identify loci where segregation followed the phenotype of avirulence on *Rps8*. From the variants obtained after filtering and imputation, only the variants for which the parental alleles were mutually polymorphic and homozygous were kept and based on the parental genotypes, alleles were coded as originating from a specific parent. Variant data for each F_2_ individual were pooled together based on the phenotype of virulence or avirulence on *Rps8*. Allelic frequencies based on the genotype called were estimated for the entire population and for each pool using VCFtools (Danecek et al., 2011). For each SNP in each pool, χ^2^ tests were performed to assess segregation distortion for the entire population and segregation bias was declared to be significant at *p* < 0.05. It was expected to find heterozygous genotypes in both pools while homozygous genotypes for each individual had to be associated to the allele from the parent carrying the same phenotype towards *Rps8*. Following subsequent oospore formation from F_2_ individuals during inoculum preparation, some heterozygous genotypes were found to acquire a stable phenotype in either the virulent or avirulent group. This was reflected in some specific individuals because this study performed individual analysis of each F_2_ progeny rather than pooled analysis as done in previous studies (MacGregor et al., 2002; Na et al., 2014; Qutob et al., 2009).

### 4.5 Selection of candidate genes from WGS data

To find potential candidate genes in the region of *Avr8*, a collection of 31 isolates previously sequenced on an Illumina Hi‐Seq (68×; Arsenault‐Labrecque et al., 2018) and from which the parents were selected was used to generate polymorphisms in the region of *Avr8*. Reads from this sequencing were aligned to *P*. *sojae* genome reference v. 3.0 using the Burrows–Wheeler transform alignment software package v. 0.7.13 (Li, 2013). Calling of SNPs and small indels was done using the Genome Analysis Toolkit, a variant‐calling pipeline based on its own best practices (DePristo et al., 2011). Larger indels and structural variations were generated using two different callers, Lumpy (Layer et al., 2014) and Manta (Chen et al., 2016). Finally, the copy number variation was detected with CNVnator (Abyzov et al., 2011). Because we expected that the causal variants leading to a phenotype of virulence toward *Rps8* would come from the virulent parent (7B) solely and we know that the isolate used to produce the reference genome (P6497) is avirulent on *Rps8*, we only kept variants from which the virulent parent isolate 7B (*avr8*) carried the alternative allele and the parent isolate 45C (*Avr8*) carried the reference allele.

### 4.6 RNA isolation and RT‐PCR

RNA isolation was performed on 7‐day‐old *P*. *sojae*‐infected soybean roots using TRIzol reagent followed by purification using the RNeasy Mini kit (Qiagen). RNA samples were treated with DNase I enzyme to avoid DNA contamination and 2 µg of each sample was used for the conversion to single‐stranded cDNA using random primers and the multiscribe reverse transcriptase from the High‐Capacity cDNA Reverse Transcription Kit (ThermoFisher Scientific). Primers for the reverse transcription PCR were designed using the PrimerQuest tool.

### 4.7 Plasmid construction

To generate *Avr3a* ^45C^ knockouts using CRISPR/Cas9, single‐guide RNAs (sgRNAs) were designed using EuPaGDT (Peng & Tarleton, 2015). The resulting sgRNAs were searched against the *P*. *sojae* genome using BLAST (Altschul et al., 1990) to check for potential off‐target sites. Based on sequence alignment, sgRNA1 and sgRNA2 were selected, targeting the *Avr3a* ^45C^ coding sequence at 90 and 139 nucleotides, respectively, from the start codon. The double‐stranded DNA fragment containing the left overlapping region, sgRNA1 or sgRNA2, coding sequences for HH ribozyme, and the right overlapping region was synthesized as a gBLOCK (Integrated DNA Technologies Inc.). The gBlock was assembled in *Nhe*I‐ and *Bsa*I‐digested plasmid pYF515 (Fang et al., 2017) using HiFi assembly (New England Biolabs). All Cas9‐sgRNA expression plasmids were sequence‐verified and named pRBCas9‐Avr3a‐1 and pRBCas9‐Avr3a‐2.

For constitutive expression of *Avr3a* ^45C^ and *Avh37* ^45C^, the coding sequences were cloned separately into the vector pUC‐57. The *Avr3a* ^45C^ and *Avh37* ^45C^ coding sequences were amplified from *P*. *sojae* isolate 45C and the Ham34 promoter was amplified from the plasmid pYF515 using specific primers. The Ham34 promoter and the coding sequences of the two candidate genes (*Avr3a* ^45C^ and *Avh37* ^45C^) were assembled in pRB‐*Avr6* digested with *Kpn*I and *Avr*II using HiFi NEB assembly (New England Biolabs). Plasmids were sequence‐verified and named pRB‐OE‐*Avr3a* and pRB‐OE‐*Avh37*.

### 4.8 Transformation of *P. sojae*

Knockout and constitutive expression vectors were introduced in *P*. *sojae* avirulent isolate 45C and virulent isolate 7B, respectively, using polyethylene glycol‐mediated transformation following a previously published protocol (Fang et al., 2017). Briefly, mycelium was grown in nutrient pea broth at 28°C in the dark. After 5–7 days, mycelium was harvested and digested using the enzyme mix to release protoplasts. Polyethylene glycol‐mediated protoplast transformation was used to introduce knockout vectors (pRBCas9‐*Avr3a*‐1 and pRBCas9‐*Avr3a*‐2) and constitutive expression vectors (pRB‐OE‐*Avr3a* and pRB‐OE‐*Avh37*). Transformants growing on regenerative medium containing 50 μg of G418 (Thermo Fisher Scientific) were selected and subcultured on V8 gellan gum medium supplemented with G418. To detect the presence of indels in *Avr3a*, genomic DNA was isolated from the stable transformants and the entire open reading frame of *Avr3a* was amplified and sequenced. For the constitutive expression of the *Avr3a* ^45C^ gene, primers M13R and M13F were used to amplify the expression cassette (promoter‐gene‐terminator) from the transformants' genomic DNA.

### 4.9 Phenotyping of *P. sojae* transformants

The virulence profile (phenotype) of selected *P*. *sojae* transformants towards *Rps8* was evaluated with the hydroponic system described previously (Lebreton et al., 2018) using cultivars PI399073a (*Rps8*) and Williams L83‐570 (*Rps3a*) and the susceptible control Haro (1‐7)1 (*rps*). The virulence phenotype towards *Rps3a* was also evaluated using Williams L83‐570 (*Rps3a*) and the susceptible control Williams (*rps rps*). Plants were scored based on a 1 to 5 scale developed by Lebreton et al. (2018), where 1 = none to limited root symptoms and 5 = mortality and advanced root necrosis. A statistical comparison (Dunnett's test) was performed to determine the phenotype of each of the isolates. When values were statistically similar to the ones obtained with cv. Haro(1‐7)1 (*rps*), the isolate was considered virulent, and when values were statistically lower, the isolate was considered avirulent.

### 4.10 Nanopore sequencing and data analysis

High‐molecular weight DNA was extracted from *P*. *sojae* isolates grown in liquid culture for 7 days with an adapted CTAB and chloroform/isoamyl alcohol method that uses a lower incubation temperature and reduced centrifugation speed to avoid DNA shearing. DNA purity was assessed with a NanoDrop spectrophotometer and quantification was performed with a Qubit 4 fluorometer. DNA libraries were prepared according to the protocol of Oxford Nanopore Technologies using the adaptor ligation kit (SQK‐LSK109). Sequencing was performed on a SpotON R9.4 flow cell using a MinION device. Sequences were base‐called with Guppy v. 3.2.4. Adapters were trimmed with Porechop (https://github.com/rrwick/Porechop) and reads were filtered for quality (Q > 7) with NanoFilt (De Coster et al., 2018). Nanopore metrics can be found in Table S3. Subsequent bioinformatic analyses comprising mapping, de novo assembly, and alignments were accomplished using the CLC Genomics Workbench v. 20 (Qiagen).

## Supporting information


**FIGURE S1** Gel image of the transcript analysis of candidate genes *Avh37* and *Avr3a* on *Phytophthora sojae* isolates with contrasting phenotypes on soybean plants carrying *Rps8*

**FIGURE S2** Sequence alignment of the *Phytophthora sojae* wild‐type isolate 45C and the stable transformant *Avr3a‐2‐3* KO obtained through CRISPR/Cas9 transformation. (a) Nucleotide sequence alignment. (b) Protein sequence alignment

**FIGURE S3** Susceptibility scores of soybean plantlets inoculated with *Phytophthora sojae* wild‐type strain and transformed isolates complementing the phenotyping assay in Figures 4–6. (a) Knockout of *Avr3a* ^45C^ in isolate 45C (complementing Figure 4). (b) Overexpression of *Avr3a* ^45C^ in isolate 7B (complementing Figure 5). (c) Overexpression of *Avh37* ^45C^ in isolate 7B (complementing Figure 6). Interactions were considered incompatible when values (indicated with a *) were significatively different from the susceptible control *rps* (Haro (1‐7)1) according to Dunnett’s test (*p* < 0.01)

**FIGURE S4** Reverse transcription PCR to detect *Avh37* ^45C^ and *Avr3a* ^45C^ transcript presence in wild‐type strain 7C and transformed strains using constitutive expression with the Ham34 promoter

**FIGURE S5** Alignment of the predicted amino acid sequences of the three different alleles of *Phytophthora sojae Avr3a* used in this study. Signal peptide, RXLR and EER motifs are shown and polymorphic residues among the different alleles are highlighted with a red background. Asterisk represent amino acid changes in *Avr3a* ^ACR12^, compared to *Avr3a* ^45C^, that leads to a recognition of the gene product by *Rps3a*, but not *Rps8* in soybean plants

**FILE S1** Consensus sequence of the *Avr3a* region from *Phytophthora sojae* isolate 45C

**TABLE S1** List of all variants found in the *Avr8* locus of *Phytophthora sojae* and carried by isolate 7B (virulent parent used for generation of the F_2_ population)

**TABLE S2** List of *Phytophthora sojae* isolates used in this study.

**TABLE S3** Metrics for Nanopore long‐read sequencing of *Phytophthora sojae* isolates

## Data Availability

The data that support the findings of this study are available in the supplementary material of this article.
