# Supplementary figures and images for: RXLR effector gene Avr3a from Phytophthora sojae is recognized by Rps8 in soybean
Source: Mol Plant Pathol. 2022 Feb 12;23(5):693–706. doi: 10.1111/mpp.13190 (PMC8995065; doi:10.1111/mpp.13190)

| Strain       | 8-3-7 | 45C | 8-5-7 | Race 7 | 2012_70 | 8-3-7 | 45C | 8-5-7 | Race 7 | 2012_70 |
|--------------|-------|-----|-------|--------|---------|-------|-----|-------|--------|---------|
| Allele Avh37 | 45C   | 45C | 45C   | 7B     | 7B      | 45C   | 45C | 45C   | 7B     | 7B      |
| Allele Avr3a | 45C   | 45C | 45C   | 7B     | 7B      | 45C   | 45C | 45C   | 7B     | 7B      |
| Rps8         | A     | A   | A     | V      | V       | A     | A   | A     | V      | V       |

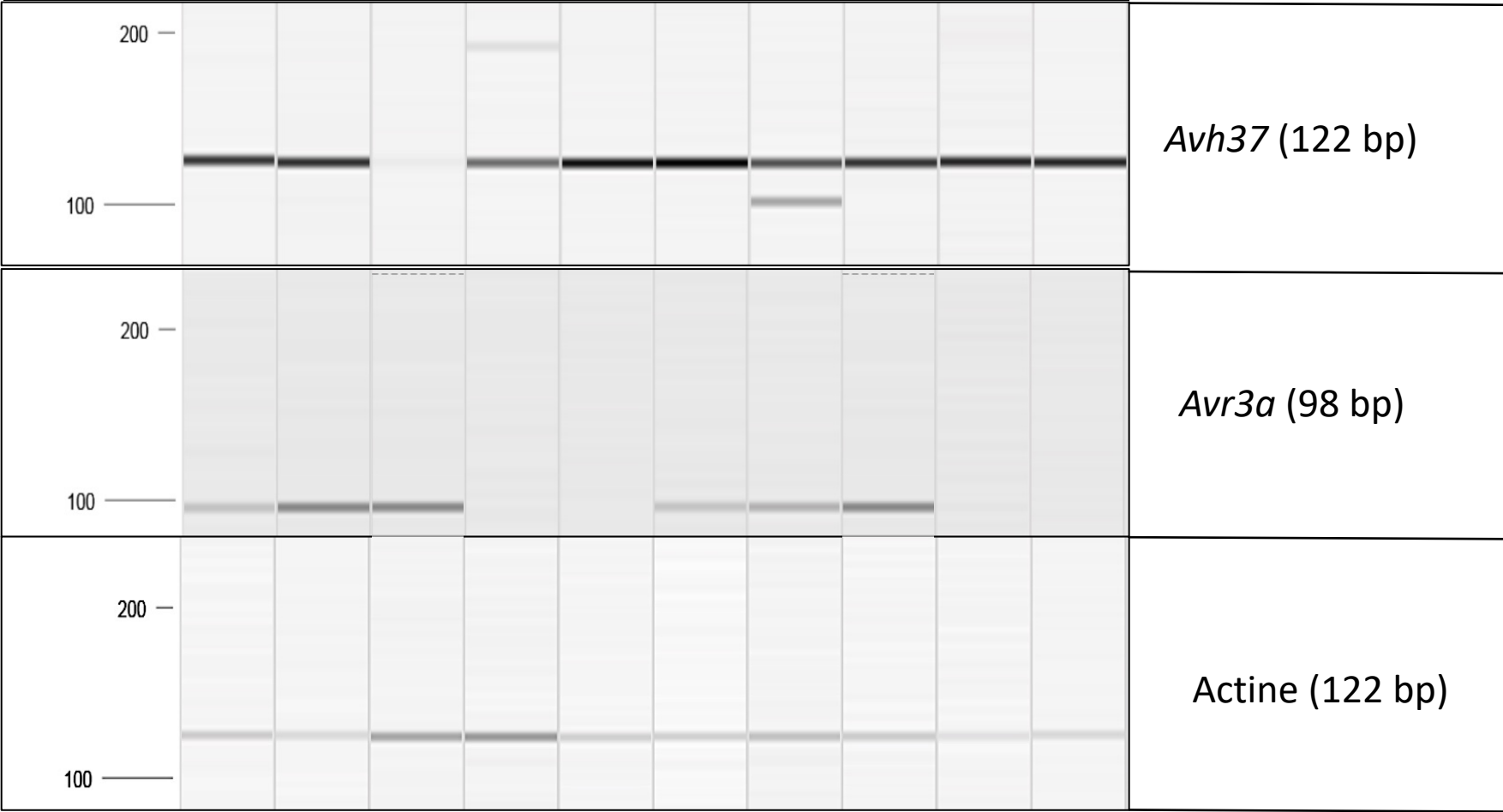

Supplement: Supplementary file 1 — FIGURE S1 Gel image of the transcript analysis of candidate genes Avh37 and Avr3a on Phytophthora sojae isolates with contrasting phenotypes on soybean plants carrying Rps8 [file MPP-23-693-s002.pdf]

(a)

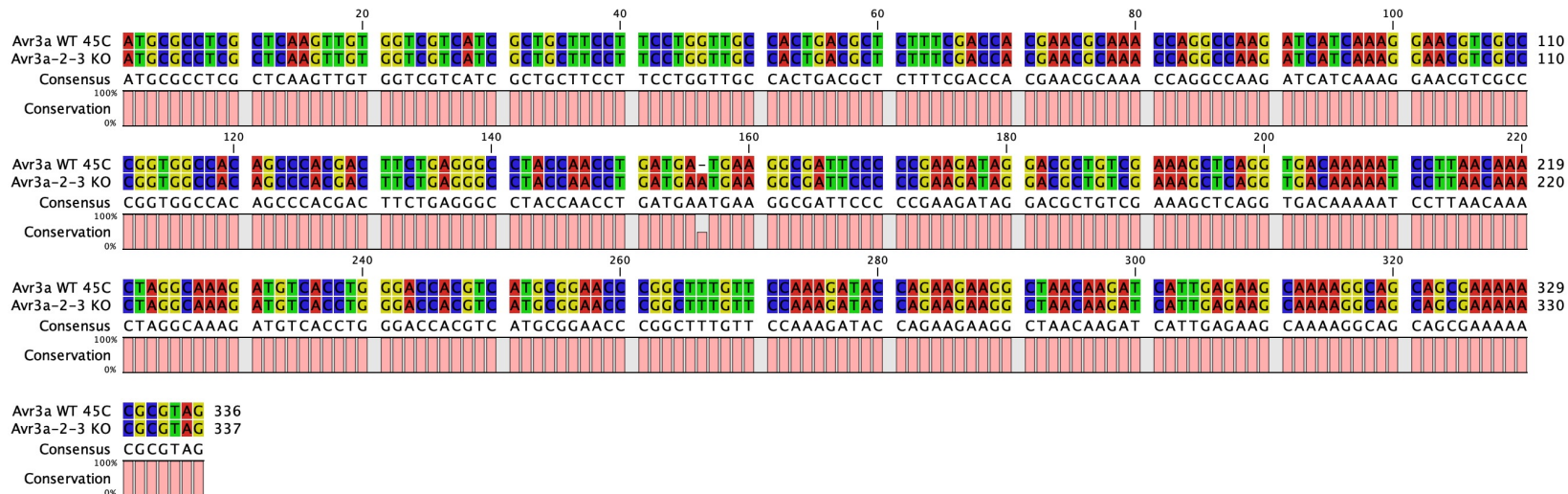

(b)

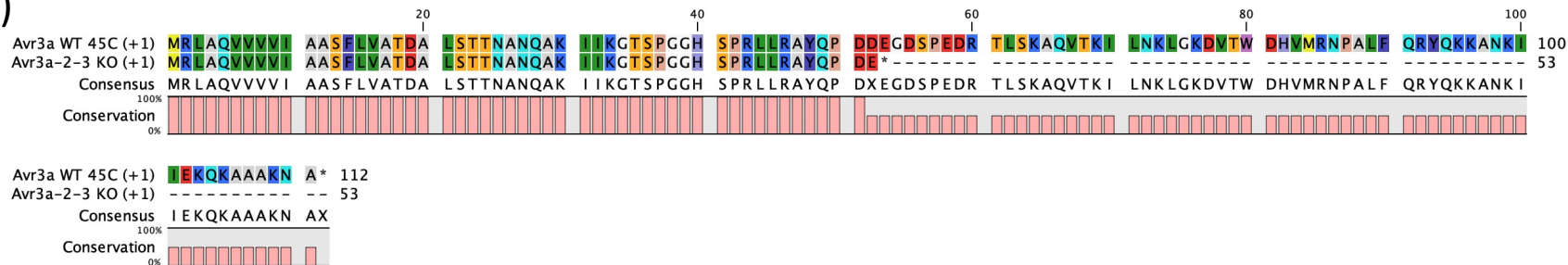

Supplement: Supplementary file 2 — FIGURE S2 Sequence alignment of the Phytophthora sojae wild‐type isolate 45C and the stable transformant Avr3a‐2‐3 KO obtained through CRISPR/Cas9 transformation. (a) Nucleotide sequence alignment. (b) Protein sequence alignment [file MPP-23-693-s009.pdf]

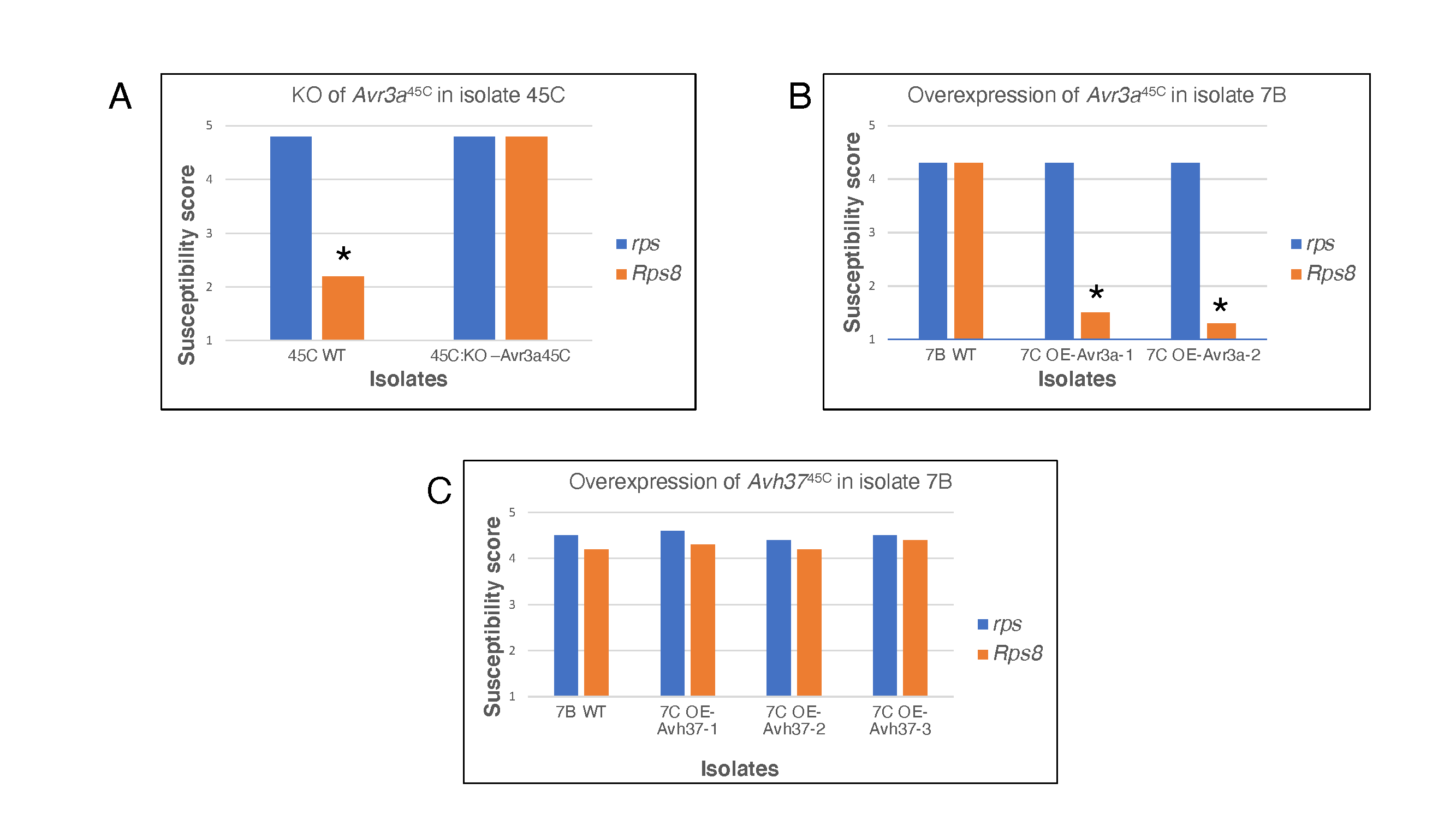

Supplement: Supplementary file 3 — FIGURE S3 Susceptibility scores of soybean plantlets inoculated with Phytophthora sojae wild‐type strain and transformed isolates complementing the phenotyping assay in Figures 4–6. (a) Knockout of Avr3a 45C in isolate 45C (complementing Figure 4). (b) Overexpression of Avr3a 45C in isolate 7B (complementing Figure 5). (c) Overexpression of Avh37 45C in isolate 7B (complementing Figure 6). Interactions were considered incompatible when values (indicated with a *) were significatively different from the susceptible control rps (Haro (1‐7)1) according to Dunnett’s test (p < 0.01) [file MPP-23-693-s007.tiff]

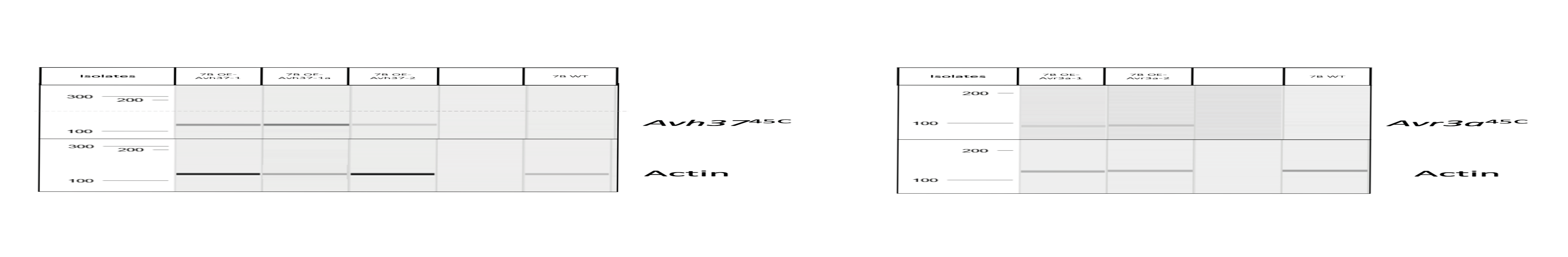

Supplement: Supplementary file 4 — FIGURE S4 Reverse transcription PCR to detect Avh37 45C and Avr3a 45C transcript presence in wild‐type strain 7C and transformed strains using constitutive expression with the Ham34 promoter [file MPP-23-693-s005.tiff]

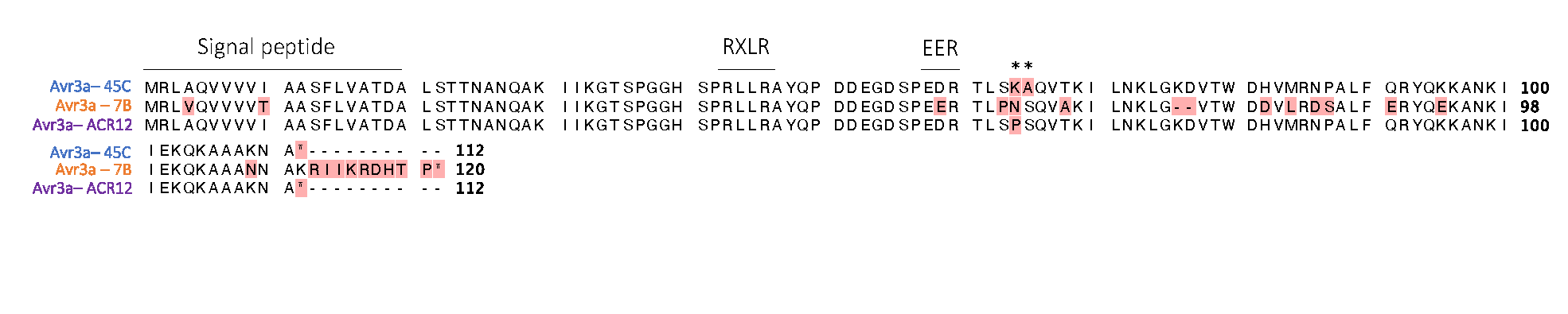

Supplement: Supplementary file 5 — FIGURE S5 Alignment of the predicted amino acid sequences of the three different alleles of Phytophthora sojae Avr3a used in this study. Signal peptide, RXLR and EER motifs are shown and polymorphic residues among the different alleles are highlighted with a red background. Asterisk represent amino acid changes in Avr3a ACR12, compared to Avr3a 45C, that leads to a recognition of the gene product by Rps3a, but not Rps8 in soybean plants [file MPP-23-693-s004.tiff]
