# Supplementary material for: RXLR effector gene Avr3a from Phytophthora sojae is recognized by Rps8 in soybean
Source: Mol Plant Pathol. 2022 Feb 12;23(5):693–706. doi: 10.1111/mpp.13190 (PMC8995065; doi:10.1111/mpp.13190)
Supplement: Supplementary file 8 — TABLE S2 List of Phytophthora sojae isolates used in this study. [file MPP-23-693-s006.docx]

**Table S2.** List of *Phytophthora sojae* isolates used in this study**.**

| **Isolate** | **Origin** | **From** | **Virulence Rps3a** | **Virulence Rps8** | **Avr3a sequence** |
| --- | --- | --- | --- | --- | --- |
| 1A | Phytophthora nursery - Ottawa | A.X | A | A | Avr3a^45C^ |
| 1C | Harrow, ON (T.A.) | A.X | A | A | Avr3a^45C^ |
| 3A | Unknown | A.X | A | A | Avr3a^45C^ |
| 3B | Fergus, ON | A.X | A | A | Avr3a^45C^ |
| 3C | Ottawa, ON | A.X | A | A | Avr3a^45C^ |
| 4A | Tayside, ON | A.X | A | A | Avr3a^45C^ |
| 4B | Mountain, ON | A.X | A | A | Avr3a^45C^ |
| 4C | Strathroy, ON | A.X | A | A | Avr3a^45C^ |
| 5A | Blenheim, ON | A.X | A | A | Avr3a^45C^ |
| 5B | Chatham-Kent, ON | A.X | A | A | Avr3a^45C^ |
| 5C | Malahide, ON | A.X | A | A | Avr3a^45C^ |
| 7A | Unknown | A.X | V | V | Avr3a^7B^ |
| 7B | Lakeshore, ON | A.X | V | V | Avr3a^7B^ |
| 7C | Mitchell, ON | A.X | V | V | Avr3a^7B^ |
| 8A | Maitland, ON | A.X | A | A | Avr3a^45C^ |
| 8B | Ottawa, ON | A.X | A | A | Avr3a^45C^ |
| 8C | Ottawa, ON | A.X | A | A | Avr3a^45C^ |
| 9A | Colbourne, ON | A.X | A | A | Avr3a^45C^ |
| 9B | Ottawa, ON | A.X | A | A | Avr3a^45C^ |
| 9C | Eagle, ON | A.X | A | A | Avr3a^45C^ |
| 22 | Ottawa, ON | A.X | V | V | Avr3a^7B^ |
| 25B | Ontario | A.X | A | A | Avr3a^45C^ |
| 25C | Lakeshore, ON | A.X | A | A | Avr3a^45C^ |
| 25D | Tilbury, ON | A.X | A | A | Avr3a^45C^ |
| 28A | Hilton, ON | A.X | A | A | Avr3a^45C^ |
| 28B | Kemptville, ON | A.X | A | A | Avr3a^45C^ |
| 28C | Ridgetown, ON | A.X | A | A | Avr3a^45C^ |
| 43 | Vars, ON | A.X | A | A | Avr3a^45C^ |
| 45A | Port Alma, ON | A.X | A | A | Avr3a^45C^ |
| 45B | Brigden, ON | A.X | A | A | Avr3a^45C^ |
| 45C | Monkton, ON | A.X | A | A | Avr3a^45C^ |
| P6497 | Mississippi, USA (B.K.) | M.G. | A | A | Avr3a^45C^ |
| P7064 | Canada (C.M.) | M.G. | V | V | Avr3a^7B^ |
| P7074 | Stoneville, MS, USA (B.K.) | M.G. | V | V | Avr3a^7B^ |
| P7076 | Stoneville, MS, USA (B.K.) | M.G. | NA | NA | Avr3a^ACR12^ |
| ACR20 | Unknown | M.G. | A | V | Avr3a^ACR12^ |
