# Supplementary material for: RXLR effector gene Avr3a from Phytophthora sojae is recognized by Rps8 in soybean
Source: Mol Plant Pathol. 2022 Feb 12;23(5):693–706. doi: 10.1111/mpp.13190 (PMC8995065; doi:10.1111/mpp.13190)
Supplement: Supplementary file 9 — TABLE S3 Metrics for Nanopore long‐read sequencing of Phytophthora sojae isolates [file MPP-23-693-s003.docx]

**Table S3.** Metrics for Nanopore long-read sequencing of *P.sojae* isolates**.**

| **Metrics** | **45C** | **7B** | **8-3-6** | **8-3-34** |
| --- | --- | --- | --- | --- |
| **# reads** | 342,548 | 254,249 | 974,874 | 1,935,588 |
| **Read length N50** | 6,437 | 17,981 | 5,612 | 4,048 |
| **Total bases** | 846,324,421 | 1,858,789,940 | 1,700,019,382 | 3,102,994,422 |
| **Longest read (and mean basecall quality)** | 134,748 (11.0) | 176,393 (8.0) | 153325 (9.4) | 118707 (12.2) |
| **Mean read quality** | 12.0 | 11.9 | 11.6 | 11.7 |
| **Q > 7** | 97.0 % | 97.8 % | 96.3 % | 96.6 % |
| **Q > 10** | 79.7 % | 81.7 % | 75.0 % | 76.3 % |
